# A Hand-Book for Nurses

**Published:** 1900-07

**Authors:** 


A Hand-book for Nurses. By J. K. Watson, M. D., Edin., Late House-Surgeon Essex and Colchester Hospital; Assistant House-Surgeon Sheffield Royal Infirmary and Sheffield Royal Hospital. American edition, under supervision of A. A. Stevens, A. M., M. D., Professor of Pathology in the Women's College of Pennsylvania, etc. Philadelphia: W. B. Saunders. 1900. [Price, $1.50.]

From the preface of Dr. Watson's book is quoted the following:

We cannot prevent nurses from attempting to acquire a certain amount of medical knowledge; and if we could, I should be no party to such a procedure. What we should rather aim at is to judiciously cater for this appetite and to use our influence to prevent the illegitimate use of such knowledge. It is common experience that the more highly qualified is the nurse the less likelihood is there of her attempting to usurp the rôle of the medical man; it is the ignorant and the badly trained nurse who thus brings discredit on her nursing-sisters.

The book admirably fulfils the purpose above indicated, that of giving a clear and satisfying amount of medical knowledge to the nurse. With a somewhat extended acquaintance with books on nursing and for nurses, the reviewer knows of none which so perfectly fits in to supply this gap in a nurse's training and a nurse's library.

